# Identity interweaving, act boundaries, illusion and reality interweaving: A study of visual narratives of scientists and citizen scientists through AI

**DOI:** 10.1371/journal.pone.0341626

**Published:** 2026-03-17

**Authors:** Yi He, Xiaoxia Jian, Weifeng Zhang, Xin Jin

**Affiliations:** 1 School of Journalism and Media, Chongqing Normal University, Chongqing, China; 2 School of Journalism and Communication, Beijing Normal University, Beijing, China; 3 Computational Communication Research Center, Beijing Normal University, Zhuhai, China; Universiti Malaya, MALAYSIA

## Abstract

The rise of generative artificial intelligence (AI) is transforming human-computer interaction, reshaping communication methods and altering public perceptions of science. This shift challenges traditional scientific authority, especially as citizen science gains prominence. While research on scientific rhetoric has focused on qualitative analyses in media, little attention has been given to how AI influences visual rhetorical narratives in science. This study employs computer visual analysis and quantitative rhetorical difference analysis to explore the intersection of AI and science through Visual Narrative Theory.It investigates the rhetorical differences between scientists and citizen scientists across three dimensions: narrate, act, and resonate. Findings reveal that both groups embody a mixed rhetoric of authority and proximity in the narrative dimension. In the act dimension, AI depicts scientists in professional roles while showing citizen scientists in practical roles, portraying scientists as “flowers in the greenhouse”. In the resonate dimension, scientists’ narratives often feature surreal elements, while citizen scientists present more everyday narratives.This analysis, utilizing computer vision and quantitative methods, offers a fresh perspective on the image of science in the AI era and suggests strategies for enhancing science communication and building trust in science using generative AI.

## Introduction

Large Language Models (LLMs), trained on vast internet-based linguistic data, are designed to produce human-like text and are increasingly transforming scientific rhetoric in the digital era. As prompt engineering expands, generative AI provides an experimental platform in which variables can be systematically manipulated through pragmatic interventions, creating new possibilities for analyzing narrative construction and communication.

Narrative remains a fundamental mode of human comprehension. Barthes highlighted its ubiquity across cultures and histories [1], while Fisher defined “narrative coherence” as intrinsically interconnected [2]. The generative capacities of AI—its ability to produce diverse structures, characters, and contexts—enable a critical reassessment of established analytical frameworks. Visual Narrative Theory (VNT) contributes by emphasizing narrative, act, and resonance as key dimensions for rhetorical inquiry beyond traditional text [3]. Concurrently, rhetorical and semiotic traditions underscore the central role of oppositionality in discourse [4,5]. The rise of citizen science, moreover, challenges professional authority and enriches our understanding of oppositional rhetorical narratives.

This study therefore explores how generative AI functions as a narrative instrument that extends rhetorical resources and reshapes AI-mediated social configurations. Such an approach advances theoretical inquiry into digital narrative construction while mapping future trajectories for rhetoric and communication research.

## Literature review

### The rhetoric of science and the rhetorical potential of artificial intelligence

The essence of rhetoric is the supplementary deployment of signs, utilizing symbolic configurations as meaningful mediators to induce cooperative behaviors and attitudes through the symbolic signification [6]. From a media perspective, rhetoric is conceptualized as a communicative mechanism that strategically disseminates information to specific audiences within particular contextual frameworks, thereby achieving persuasive or motivational objectives [7].Scientific rhetoric should definitively establish constructive categories emerging from authentic scientific discursive practices, simultaneously providing analytical frameworks that elucidate how these discourses are generated and subsequently evaluated as scientifically legitimate. The “art of rhetoric” and its foundational principles fundamentally serve to enable scientists in substantiating their claims, empowering audiences to critically assess their rational validity, and consequently facilitating decision-making processes grounded in these discursive representations [6], which intrinsically encompass scientific comprehension and epistemological trust.

Science necessitates rhetoric, situating scientific discourse within specific cultural contexts and delineating the permissible boundaries of scientific dialogue. The critical aspect of scientific rhetoric transcends quotidian linguistic frameworks, instead interpreting from identity construction [8]. For example, scientists construct an image of themselves as authoritative through a distinctive rhetorical narrative style, thereby performing “boundary work” that differentiates science from non-science [9].How scientists construct style and content to enhance or protect the ideology of their professional authority [9], discursive and rhetorical techniques have been identified as key to constructing scientists’ discursive authority because of their “unqualified public trust” and “exclusive legitimacy” [10,11]. This manifests through scientists’ hierarchical communicative postures when addressing public audiences [12], scientific communication texts that emphasize disciplinary gravitas [13], and strategic linguistic demarcations using first-person pronouns to distinguish qualified scientific communicators [14]. Previous research has shown that communication among scientists has evolved toward heightened specialization, with rhetorical practices often exhibiting a closed nature: characterized by the use of specialized vocabularies, graphs, charts, and formulas, as well as data-driven and theory-driven arguments [7].Since the late twentieth century, scientific popularization has proliferated within cultural markets, necessitating compelling narrative strategies, engaging protagonists, and aesthetic sophistication to captivate public interest and render scientific subjects both relevant and intellectually appealing. Science has been rhetorical from the beginning, or in Gross’s vision, subject to a “comprehensive” rhetorical interpretation [6].

Currently, artificial intelligence rarely serves as a subject of rhetorical inquiry, which may be due to the immaturity of AI technologies or its limited prevalence. However, with the advancement of AI, it is poised to become one of the many personal, social, and technological features shaping our digital landscape, much like social media [15]. Certain AI attributes are unequivocally rhetorical, fundamentally originating from algorithmic networks constructed through human epistemological frameworks. Generated content exemplifies this phenomenon, particularly evident in social media contexts where manipulative actors strategically exploit algorithmic mechanisms and computational propaganda to distort public sentiment [16]. Scholarly investigations into AI’s rhetorical dimensions, rooted in critical algorithmic studies and rhetorical pedagogical methodologies, promise substantive contributions to existing rhetorical discourse. Despite AI’s inherent limitations in comprehending intentionality, worldviews, or psychological nuances, humans demonstrate a propensity to deploy these technologies disingenuously—instrumentalizing them to amplify biases, generate misinformation, and propagate false narratives [17]. Consequently, AI technologies potentially compromise individual agency, employing biased rhetorical discourses that systematically disenfranchise marginalized populations. Emerging research has examined AI-generated writing through genre-specific lenses, using GPT technologies to conduct comparative analyses between AI and human-generated textual productions.Notably, AI-generated texts exhibit first-person narrative strategies and demonstrate heightened affective valence [18]. Irrespective of evaluative judgments, artificial intelligence has firmly established its presence within contemporary communicative ecosystems. For scholarly practitioners, comprehending how AI simulates linguistic phenomena—thereby recursively transforming communicative practices—has become imperative. This emerging domain not only presents novel research trajectories but also demands innovative methodological approaches to rhetorical investigation [15].

### Rhetoric, narrative, and the visual narratives of science

Narrative is rhetoric [19]. The theories of Kenneth Burke [20]and Wayne Booth [21] highlight that narrative serves as a unique and powerful means for authors to convey knowledge, emotions, values, and beliefs to their audience. Indeed, viewing narrative as a means of conveying knowledge, emotions, values, and beliefs is to consider narrative as rhetoric [22].Narratives within rhetorical discourse indicate that rhetorical texts are crafted to induce social action among the audience. These discourses often utilize narrative elements as a means to achieve their argumentative, persuasive, or other motivational objectives. The study of narratives within rhetorical discourse primarily addresses the role of narrative in rhetorical communication [23].Narratives within rhetorical texts possess cognitive and persuasive functions, influencing individuals’ understanding of the world, capturing audience attention, and fostering a sense of identification with the audience [24].

Rhetoric often focuses on the study of texts, viewing “rhetoric” itself as a form of textual science that provides a comprehensive theoretical and methodological framework for text analysis [25]. “Science” is certainly regarded as a form of textual culture, characterized by varying communicative objectives, diverse textual traditions, distinct genres, different concepts of truth, and various discourse compositions related to types of social interaction [26], as scientific texts are also socially or linguistically constructed.Textual rhetoric engages in “identity work,” examining how various works establish textual roles and positions for different participants, as well as the relationships among them [8]. Rhetoric thus defines the gap in understanding between scientists and non-scientists—or so-called “ordinary people”—regarding the same world [8], portraying ordinary individuals as irrational beings who are easily manipulated and exploited.Visual narrative provides an important theoretical basis for comprehending visual rhetorical discourse, as images can serve as sources of narrative [27,28] and are more efficient in processing information than text-based narratives [29].Within scientific communication paradigms, visual imagery transcends mere informational representation, emerging as a sophisticated rhetorical boundary-demarcation strategy. AI-generated scientific images accentuate this rhetorical tendency, as AI—similar to social media—is becoming a key personal, social, and technological force shaping the digital landscape [15].

To begin with, the three dimensions of visual narrative—Narrate (visual complexity, background, composition, color), Act (people, objects), and Resonate (realism, dynamism, and taboo)—provide a multi-layered rhetorical mechanism for constructing scientific boundaries [3]. Through carefully designed composition, color, and expressions of realism, scientific images can reinforce the boundaries between professional science and public science. For example, rigorous mapping styles, precise data visualizations, and professional image aesthetics serve as visual markers that distinguish “professional” from “non-professional” scientific expressions.

Since the theory of visual narrative primarily originates from the fields of advertising and commercial communication, its dimensions do not fully reflect the unique context of scientific communication, particularly in the rapidly evolving landscape of artificial intelligence. Scientific communication is not merely about the transmission of information; it is a complex socio-cultural practice that involves knowledge production and the construction of authority. Therefore, this study aims to supplement the original dimensions of Visual Narrative Theory based on the characteristics of scientific communication and the technological innovations brought by generative artificial intelligence. Here, we propose the following research questions:

RQ1: How can the dimensions of Visual Narrative Theory be expanded in the context of artificial intelligence and scientific communication to better accommodate the rhetorical study of scientific narratives?

Furthermore, considering that research on the rhetoric of science tends to focus more on rhetorical strategies in texts, these rhetorical narrative strategies serve as crucial criteria for delineating boundaries with non-scientists, emphasizing the authority of science and persuading the public through professional and authoritative rhetorical narratives that compel understanding and trust in science. However, such rhetoric often tends to be closed; when science aims to be “understood by the public,” it must become accessible and engaging to facilitate participation. Since the late 20th century, popular science has flourished in the cultural marketplace, requiring captivating narratives, engaging protagonists, and appealing aesthetics (and sound, when applicable) to attract readers’ interest while making the subject relevant and aesthetically appealing [7].

Due to the nature of scientific research, many have developed the notion that scientific inquiry is a domain involving only a few professional scientists [30,31]. This is not the case; citizen science is on the rise, particularly as social media provides significant pathways for its dissemination, resulting in the number of citizen scientists surpassing that of professional scientists [32,33]. However, Aristotle’s rhetorical triangle theory, which categorizes rhetoric into mutually exclusive modes of persuasion (emotional, ethical, and logical appeals) [34], faces challenges with the emergence of citizen science projects, as many citizen science initiatives often employ a mix of persuasive approaches in their narratives, resulting in hybrid rhetoric [35]. Previous studies on narrative rhetoric may not suffice for exploring the boundaries between science and citizen science. As a typology, visual narrative provides a theoretical foundation for exploring hybrid visual rhetoric. The development of artificial intelligence, much like the rise of social media in the past, endows rhetoric with new potentials. Therefore, this study poses the following research question:

RQ2: How does artificial intelligence construct the visual narrative rhetoric of scientists and citizen scientists? How does this narrative construction influence the public perception of science?

## Methods

### Data collection and pragmatic experiments in artificial intelligence

#### Proposing the concept of pragmatic experiments.

Pragmatics emphasizes that language derives meaning within contextual structures, functioning not only as a medium of information transfer but also as a mechanism for shaping cognition and constructing interpretive frameworks [36,37]. With the rise of generative artificial intelligence, these theories gain renewed relevance, particularly as prompt-driven outputs demonstrate how linguistic cues guide models toward specific representational outcomes [38].

Semiotics further provides a structured framework for understanding how prompts operate. By distinguishing surface-level signs from deeper symbolic meanings, semiotics explains how linguistic inputs act as cognitive triggers that enable AI models to convert abstract language into concrete visual representations. This mechanism reduces randomness and foregrounds intentionality within generative processes [39], while simultaneously revealing the cultural encodings and social imaginaries embedded within model training data [40].

Building on these foundations, this study proposes the concept of a “pragmatic experiment.” Unlike conventional prompt engineering, which primarily focuses on optimizing outputs, a pragmatic experiment constitutes a research method designed to examine how generative AI constructs visual meaning systems under controlled contextual conditions [41]. It seeks to uncover AI’s rhetorical operations—how it interprets symbolic cues, reproduces cultural schemas, and amplifies or transforms human cognitive stereotypes [40,42]. Thus, pragmatic experimentation is not a technical procedure but a theoretical and methodological framework for studying AI-mediated visual signification.

#### Using prompt words in pragmatic experiments to control research variable errors.

Current studies demonstrate that generative AI in image production tends to amplify existing racial and gender stereotypes when prompts involve occupations or social roles. This is evident in the predominance of White representations [43], the absence of minority groups [44], and the underrepresentation of women in male-dominated professions alongside their overrepresentation in female-dominated ones [45]. These findings highlight that AI-mediated content creation inherently carries national, racial, and gender biases..

This study adopts a pragmatic experimental approach, which not only emphasizes isolating and controlling variables under standardized conditions [46], but also treats prompts as a form of linguistic-semiotic intervention to test differences in how AI constructs visual boundaries between scientists and citizen scientists. To mitigate the pragmatic orientation of “prompt engineering” and its potential influence on conclusions about visual boundary construction, the study designs two experimental groups: concept-free prompts and concept-driven prompts. This dynamic pragmatic experimental method ensures that conceptual prompts do not bias the results.

The experiment follows a factorial design of (conceptual × non-conceptual) × (gendered × non-gendered) × (scientist × citizen scientist). Based on the DALL·E 3 visual model accessed through the GPT API, the dataset was collected as follows: in the concept-free prompt group, for non-gendered prompts, two images were generated per country across both scientists and citizen scientists, yielding 772 images. For gendered prompts, one male and one female image were generated per country across both groups, yielding another 772 images. In total, the concept-free prompt group produced 1,544 images. The concept-driven group followed the same logic, resulting in an additional 1,544 images. Altogether, 3,088 images were collected. The definitions of “scientist” and “citizen scientist” were derived from prior scholarship [14,47–50], with prompt engineering details for both conceptual and non-conceptual approaches presented in Table 1.

**Table 1:** Prompt engineering.

	scientist	Citizen scientist
**Non-Conceptual**	Generate a typical, lifelike image of a scientist from {country}, as realistic and human-like as possible.	Generate a typical, lifelike image of a citizen scientist from {country}, as authentic and human-like as possible.
**Conceptual**	Scientists are professional individuals dedicated to scientific research, exploring the underlying laws of natural and social phenomena. They possess a systematic scientific knowledge background and, through methods such as observation, experimentation, and theoretical derivation, are committed to discovering new knowledge, developing novel theories, or solving scientific challenges, thereby advancing scientific progress and technological innovation. Based on this definition of a scientist, create a single, realistic portrait representative of a scientist from {country}.	Citizen scientists are ordinary individuals without formal scientific backgrounds who possess recognized scientific literacy and actively engage in the processes of scientific research, knowledge production, and science communication. Although they lack professional training, their participation plays a significant role in advancing science and enhancing public understanding of scientific issues. Generate a typical, lifelike image of a citizen scientist from {country}, as realistic as possible, featuring only one individual, based on the above definition.

This study does not directly measure public perceptions of scientific imagery; rather, it employs pragmatic experimentation to analyze how AI, driven by language, rhetorically constructs the boundaries of science. By manipulating prompts, symbolic interventions, and contextual variables, the experiment reveals how AI uses language and symbols to generate visual narratives. This approach not only helps to clarify AI’s potential rhetorical role in science communication but also minimizes research bias. Thus, pragmatic experimentation serves both as a tool for variable control and prompt-effect validation, and as an experimental methodology for examining AI’s rhetorical capacity and exploring how the interaction among language, symbols, and technology shapes the boundaries of scientific narratives.

### The expansion of visual narrative theory in science communication and visual coding rules

To address RQ1, this study adapts research on science communication within the framework of Visual Narrative Theory, with particular attention to representations of scientists and citizen scientists. The dimensions of visual narrative are expanded to reflect these research subjects.

#### Narrative dimension.

In the narrative dimension, background enhances persuasiveness. Scientific presence is typically linked to laboratories [51] or natural settings [52].and here is defined as “presence,” highlighting the embodied, practical nature of science. Laboratory rigor, precise instruments, and field observations reinforce authenticity and credibility, strengthening the connection between theory and practice. Chambers [53] noted “Eureka!” as a title indicating scientific discovery, illustrating how textual annotations complement visual content. Narratives combine linguistic and visual modes to aid recall and guide reasoning [54].Because images lack explicit argumentation, visual persuasion theory stresses the need for textual elements to convey meaning [55]. Text can guide audience attention, provide context, and clarify emotional or action-related backgrounds, thereby enhancing the coherence of visual narratives. In this sense, text functions as a supportive tool within the narrative dimension of Visual Narrative Theory. Similarly, visual structures such as shot type, composition, and angle operate as rhetorical strategies of power and accessibility. For instance, central composition often conveys authority—echoing historical depictions of emperors positioned at the center and viewed from above—whereas close framing emphasizes intimacy and accessibility [56].

Visual representations of science often rely on signs—symbols such as molecular models or equations [57,58]. Research shows that self-images of Greek scientists contained fewer such symbols [59]. Generative AI may similarly employ scientific symbols to depict scientists and citizen scientists. These knowledge symbols are not static markers but active tools that convey information, shape relationships, and guide thought through their logical combinations. They can trigger specific scientific cognition and behaviors, consistent with the “act” dimension, where “objects can be agents.” Thus, it is reasonable to extend the act dimension by including such symbolic objects.

#### Act dimension.

In the act dimension, incorporating age, visibility, emotional characteristics, as well as attire and props into the actions of scientists aims to emphasize the significant roles these factors play in shaping the professional image of scientists and the process of scientific exploration. Age reflects the accumulation of experience and knowledge; visibility influences public perception and interest in science; emotional characteristics showcase the passion and dedication of researchers; and attire and props not only signify professionalism but also support their research activities. Together, these dimensions form the core characteristics of science, highlighting the diversity and complexity of scientific pursuits.

#### Resonate dimension.

Research indicates that photography lacks narrative capacity. Compared to film—a medium originally referred to as “animated photography”—photography faces challenges in addressing issues of time, narrative, and fiction. When photography is viewed as capturing snapshots, the technology itself almost inevitably resists the capture of the passage of time; there is no time elapsed because photography, much like the law, struggles to incorporate fictional elements, which are well-known as significant generators of narrative [27]. However, modern technology can imbue photography with additional elements, including fiction and time [60]. Fluidity is a dynamic meaning-making process that transcends static visual representation. In image generation, fluidity manifests as a continuous transformation of temporal and spatial elements, resulting in images that exhibit multidimensional, nonlinear characteristics. This fluidity can be specifically reflected in the dimension of time: images are no longer static captures of a single moment but instead showcase the dynamic process of time passing; in terms of spatial transformation, the boundaries of images become fluid, and the relationships between different elements are in a state of continuous reorganization. Previous visual narratives did not address the description of fluidity, and research has categorized it within the dynamism dimension of resonate. In addition, since the gaze of the figure represents the interaction with the public, and the movement emphasizes the dynamic interaction between the figure and other objects in the image, they both contribute to the vividness of the image [56] and are therefore classified in the Resonate dimension.

Furthermore, in the extended visual narrative framework, images were coded for “taboo” and “surrealism.” Surrealism was further divided into “realistic” and “surreal” [3]. Taboo is used here as an analytical category to capture visual elements that transgress social norms or cultural expectations, or evoke discomfort and moral tension. In the context of science communication, this dimension is particularly relevant because many scientific debates—such as those concerning genetic modification, human experimentation, or environmental risks—carry a strong taboo component. Coding for taboo thus allows us to identify how AI-generated images visually frame the controversial and ethically sensitive aspects of science.

**Fig 1:**
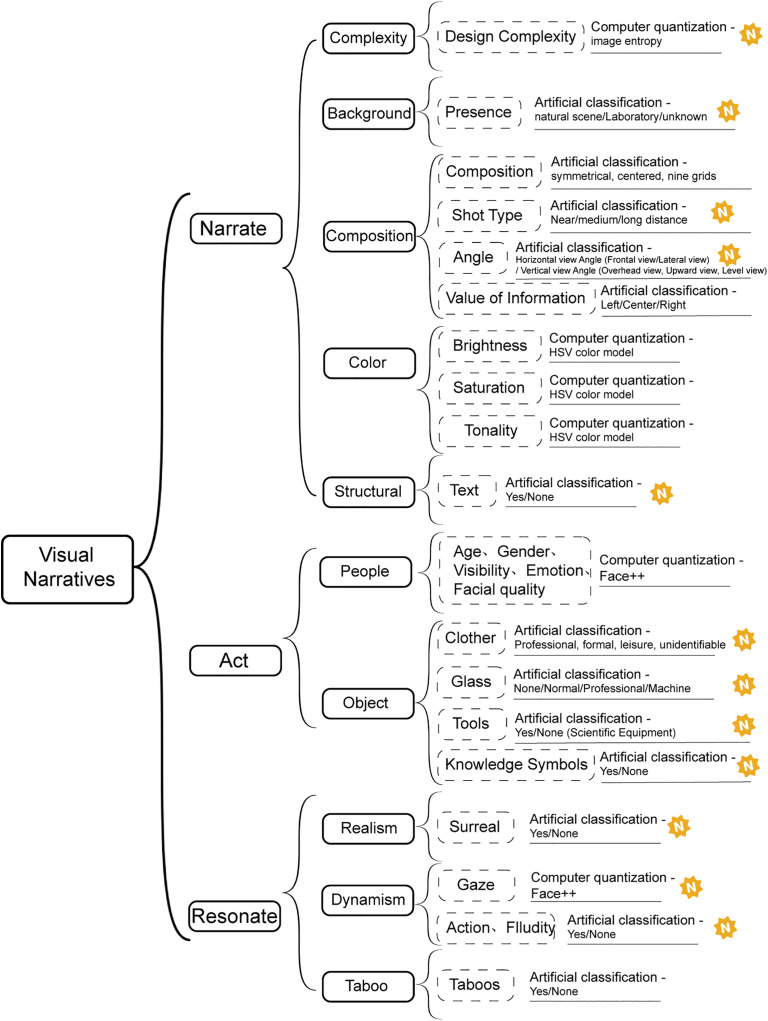
Dimensions of visual narrative theory coding.

To sum up, due to the large number of variables, the coding dimension of visual narrative is presented in a frame structure diagram (Fig 1), which is carried out in a mixed coding method of manual and computer. The logo marked with “N” represents the newly added dimension or indicator, as well as the new method.

### Image feature quantification and coding rules

The visual features of AI-generated images were quantified using Python’s OpenCV library by extracting grayscale and gradient-magnitude histograms. All images were standardized to 256 × 256 pixels. For each image, grayscale and gradient histograms were converted into feature vectorsF={f1, f2,....., fn}, where each feature $f_{i}$ was represented by an m-bin normalized histogram describing pixel intensity or edge characteristics.

The information content of each feature was measured using Shannon entropy:


$$\begin{array}{c} {\text{H}}_{\text{i}}=-\sum {\mathrm{\backslash nolimits}}_{j=\text{1}}^{m}p_{ij}\text{log}{(p}_{ij}) \end{array}$$ (1)

where $p_{ij}$ denotes the normalized value of feature $f_{i}$ in bin j, with $\sum_{j=\text{1}}^{m}p_{ij}=\;\text{1}$ 。Higher entropy indicates greater feature variability.

To ensure comparability across features, entropy values were normalized to obtain feature weights:


$$W_{i}=\;\frac{H_{i}}{\sum_{i=\text{1}}^{n}H_{i}}$$ (2)

where n is the total number of extracted features.

Color features, including brightness, saturation, and hue, were extracted in the HSV color space. Average hue values were computed and used to categorize images according to defined HSV ranges. For scientific task-specific features such as facial quality, gaze, and age, the Face++ API was employed for recognition and analysis.

To verify the reliability of manual coding, eight pragmatic prompt experiment groups were sampled, covering all combinations of concept presence, gender, and subject type:


(Concept×No Concept)×(Gender×No Gender)×(Scientist×Citizen Scientist)

For each group, 20 images were randomly re-coded. The overall Cohen’s Kappa was 0.860, with an average similarity rate of 0.945, meeting the standard for reliability.

### Ethics statement

This study did not involve human participants or identifiable personal data. All analyzed images were generated by artificial intelligence models under controlled experimental conditions. Therefore, ethical approval and informed consent were not required.

## Results

### Descriptive statistical analysis

The number of images generated by AI for both scientists and citizen scientists is 1,544. To gain an initial understanding of the overall differences in visual narratives between scientists and citizen scientists, descriptive statistical analysis has been conducted on the entire dataset.

**Fig 2:**
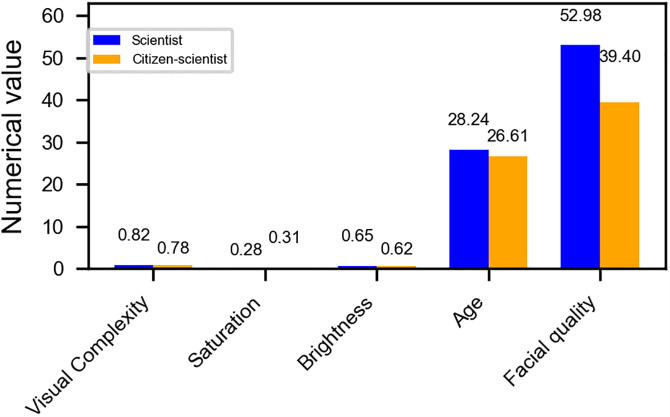
Comparison of image numerical characteristics between scientists and citizen scientists.

In the numerical data (Fig 2), in the dimension of design complexity (with a maximum value of 1), the average value of scientists is 0.82, slightly higher than that of citizen scientists at 0.78. This indicates that in the images generated by AI, the visual presentation of scientists becomes more complex, which may reflect the symbolic expression of their professionalism and rigor. In terms of color saturation, the score of scientists was 0.28, and that of citizen scientists was 0.31. Both are at a relatively low level. In terms of brightness, the score of the scientist’s image was 0.65, slightly higher than that of the citizen scientist at 0.62, with a gap of 0.03. In terms of age, the two groups are extremely close. The age of the scientist is 28.24 years old, and that of the citizen scientist is 26.61 years old, with a difference of only 1.63 years. This reflects that “youthfulness” is one of their common characteristics. The most significant difference was in facial quality (i.e., image clarity and recognition), with scientists scoring 52.98, much higher than citizen scientists’ 39.4, and the gap was 13.58 points.

**Fig 3:**
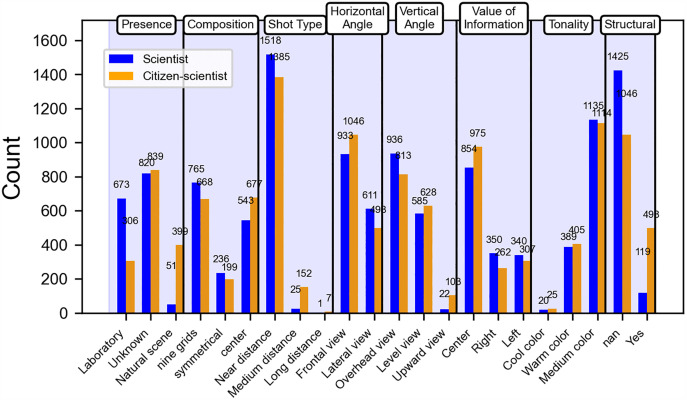
Comparison of narrative dimension quantities between scientists and citizen scientists.

In the narrative dimension (Fig 3), the number of scientists present in laboratory settings (673) is significantly greater than that of citizen scientists (306). Conversely, in natural scenes, the number of citizen scientists (399) greatly exceeds that of scientists (51). Regarding images without text annotations, the number of scientists (1,425) is also higher than that of citizen scientists (1,046). In terms of centered composition, medium-distance framing, overall eye level, and intermediate information value classification metrics, the number of citizen scientists slightly exceeds that of scientists. In contrast, for grid composition, close-distance framing, overall side view, and overall low-angle classification metrics, the number of scientists is somewhat greater than that of citizen scientists. In the remaining classification metrics, the difference between the number of scientists and citizen scientists is minimal.

**Fig 4:**
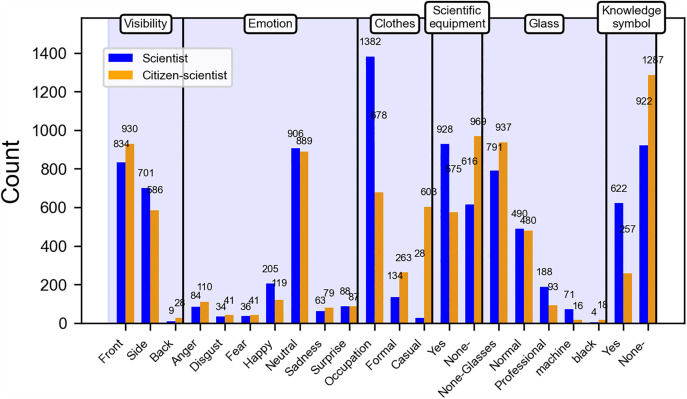
Comparison of act dimension quantities between scientists and citizen scientists.

In the act dimension (Fig 4), the number of scientists wearing professional attire (1,382) is significantly greater than that of citizen scientists (678). Conversely, the number of citizen scientists in casual clothing (603) far exceeds that of scientists (28). When it comes to the presence of scientific tools, the number of scientists (928) is also much higher than that of citizen scientists (575). Additionally, the number of scientists displaying knowledge symbols (622) is significantly greater than that of citizen scientists (257). In terms of visibility from the side, expressions of happiness, and professional glasses classification metrics, the number of scientists slightly exceeds that of citizen scientists. Conversely, in terms of front visibility, formal attire, and no glasses classification metrics, the number of citizen scientists is slightly greater than that of scientists. In the remaining classification metrics, the difference in numbers between scientists and citizen scientists is minimal.

**Fig 5:**
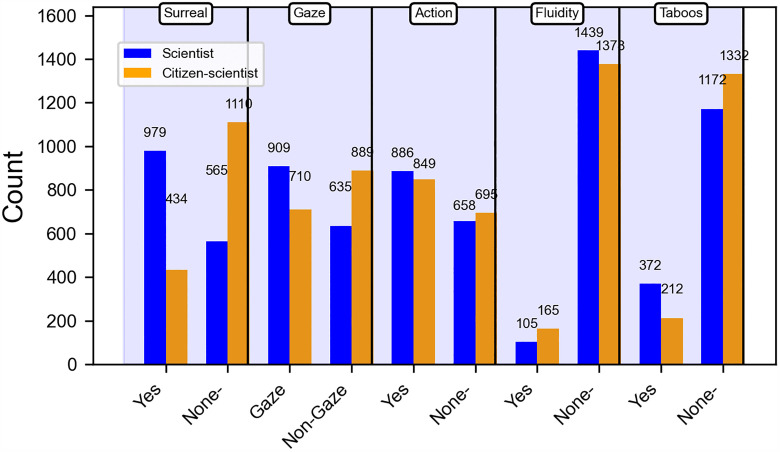
Comparison of resonate dimension quantities between scientists and citizen scientists.

In the resonate dimension (Fig 5), the number of scientists in surreal contexts (979) is significantly greater than that of citizen scientists (434). Similarly, the number of scientists depicted in gazes (909) far exceeds that of citizen scientists (710), and the number of scientists in grotesque representations (372) is also much higher than that of citizen scientists (212). In the remaining classification metrics, the difference in numbers between scientists and citizen scientists is minimal.

### Differential analysis

To address RQ2, the study examines the overall visual narrative differences between scientists and citizen scientists based on descriptive statistical analysis. Different types of data were analyzed using the following methods:

1. Continuous Data: For continuous variables (such as design complexity, brightness, color saturation, face quality, etc.), the data normality is first evaluated through the Shapiro-Wilk test, and the homogeneity of variance is verified through the Levene test. It conforms to the normal distribution and homogeneity of variance test. Therefore, the independent sample t-test is used to compare the overall performance of scientists and citizen scientists.
2. Categorical Data: The proportion of cells with an expected frequency of less than 5 in the categorical variable data does not exceed 20%. Therefore, a chi-square difference test is conducted to assess the distribution differences in categorical variables between the two groups.

Subsequently, to examine the differences in more granular categorical indicators, standardized residual analyses were conducted for those chi-square tests that yielded significant categorical data. Typically, a residual greater than 2 indicates a significant difference in that “subcategory”. Furthermore, in order to be integrated and displayed in one graph, both the independent sample t-test and the standardized residual data are presented in one graph, and their significance is (p < 0.001). During the statistical analysis, comparisons were made between scientists and citizen scientists, where positive values indicate a numerical advantage for scientists and negative values indicate a numerical advantage for citizen scientists.

To reduce the risk of inflated Type I errors resulting from multiple comparisons, all p-values were adjusted using the Benjamini–Hochberg false discovery rate (FDR) correction. All reported significant results remained significant after correction.

**Fig 6:**
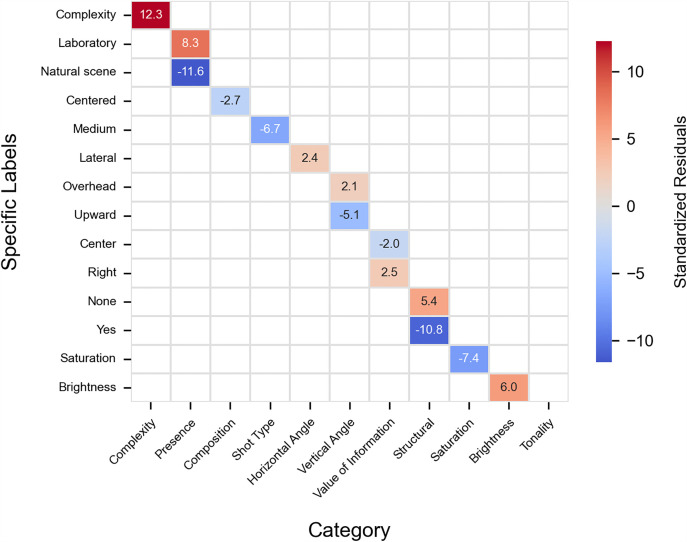
Significance indicators of narrative dimensions. This heatmap displays the standardized residuals of tertiary visual indicators (y-axis) within their secondary categories (x-axis) in the narrative dimension. Positive values (warm colors) indicate greater prevalence in scientist images, while negative values (cool colors) indicate greater prevalence in citizen scientist images. Color intensity reflects the magnitude of deviation. Overall, visual complexity, laboratory settings, lateral and overhead viewpoints, right-side information value, and higher brightness are more common in scientist images, whereas natural scenes, centered composition, medium-distance framing, low-angle views, centered information value, higher color saturation, and structured text annotations occur more often in citizen scientist images.

In the narrative dimension, the significance indicators chart highlights the significant categorical metrics (Fig 6). In the images of scientists generated by AI, the following attributes exhibit a significant numerical advantage: complexity (t = 12.27, p < 0.001), laboratory settings (r = 8.29, Cramer’s V = 0.36, p < 0.001), overall lateral view (r = 2.40, p < 0.001), overhead view (r = 2.09, Cramer’s V = 0.14, p < 0.001), right-side information value (r = 2.52, Cramer’s V = 0.09, p < 0.001), brightness (t = 5.95, p < 0.001), and lack of text annotations (r = 5.39, Cramer’s V = 0.30, p < 0.001).

In contrast, in the images of citizen scientists generated by AI, attributes with a higher numerical advantage include the presence in natural scene (r = −11.60, Cramer’s V = 0.36, p < 0.001), centered composition (r = −2.71, Cramer’s V = 0.09, p < 0.001), medium-distance framing (r = −6.75, Cramer’s V = 0.18, p < 0.001), up-angle view (r = −5.21, Cramer’s V = 0.14, p < 0.001), centered information value (r = −2.00, Cramer’s V = 0.09, p < 0.001), color saturation (t = −7.43, p < 0.001), and the presence of structured text annotations (r = −10.79, Cramer’s V = 0.30, p < 0.001).

**Fig 7:**
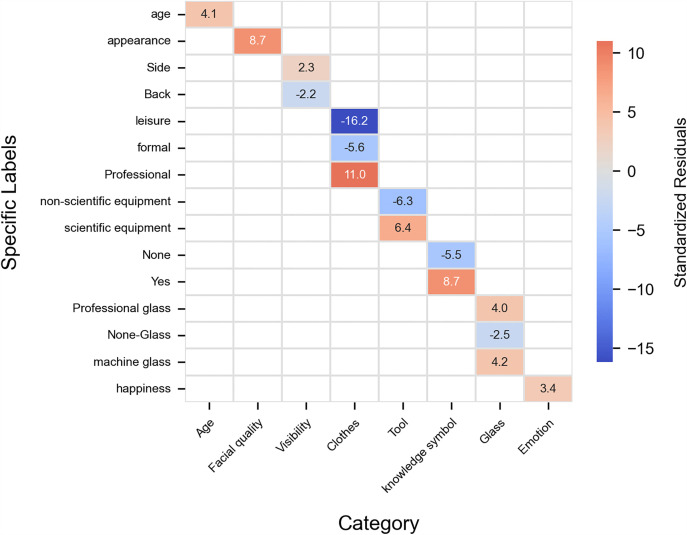
Significance indicators of act dimensions. This heatmap shows the standardized residuals of tertiary visual indicators (y-axis) within their secondary categories (x-axis) under the Narrative Dimension. Positive values (warm colors) indicate greater prevalence in scientist images, while negative values (cool colors) indicate greater prevalence in citizen scientist images. Visual complexity, laboratory settings, lateral and overhead viewpoints, right-side information value, and higher brightness appear more often in scientist images, whereas natural scenes, centered composition, medium-distance framing, low-angle views, centered information value, higher color saturation, and structured text annotations are more common in citizen scientist images.

In the act dimension, the significance indicators chart highlights the significant categorical metrics (Fig 7). In the images of scientists generated by AI, the following attributes exhibit a significant numerical advantage: age (t = 4.10, p < 0.001), facial quality (t = 8.65, p < 0.001), side profile (r = 2.27, Cramer’s V = 0.09, p < 0.001), expressions of happiness (r = 3.38, Cramer’s V = 0.10, p < 0.001), professional clothes (r = 10.97, Cramer’s V = 0.52, p < 0.001), presence of scientific tools (r = 6.44, Cramer’s V = 0.23, p < 0.001), and display of knowledge symbols (r = 8.71, Cramer’s V = 0.26, p < 0.001).

In contrast, in the images of citizen scientists generated by AI, attributes with a higher numerical advantage include being back-facing (r = −2.21, Cramer’s V = 0.09, p < 0.001), leisure clothes (r = −16.19, Cramer’s V = 0.52, p < 0.001), formal clothes (r = −5.56, Cramer’s V = 0.52, p < 0.001), absence of scientific tools (r = −6.27, Cramer’s V = 0.22, p < 0.001), and absence of knowledge symbols (r = −5.49, Cramer’s V = 0.26, p < 0.001).

**Fig 8:**
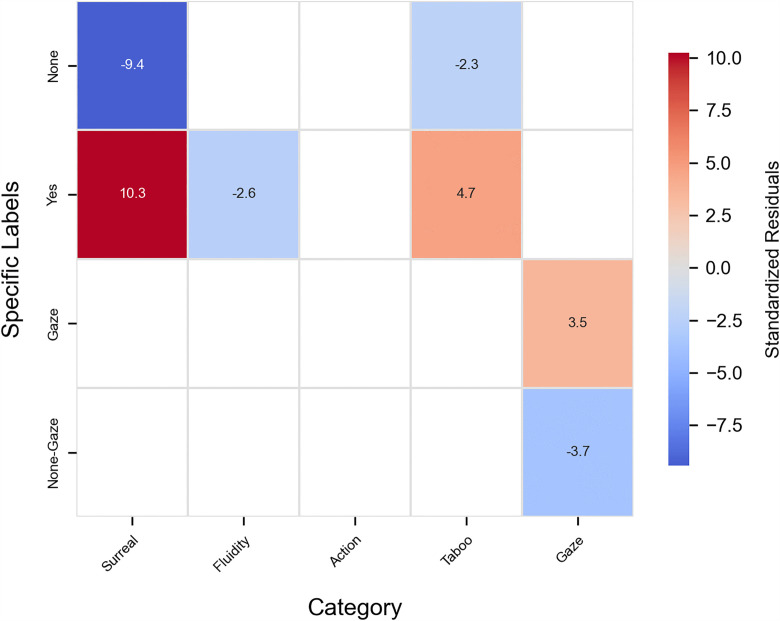
Significance indicators of resonate dimensions.

In the resonate dimension, the significance indicators chart highlights the significant categorical metrics (Fig 8). In the images of scientists generated by AI, the following attributes exhibit a significant numerical advantage: surreal elements (r = 10.25, Cramer’s V = 0.35, p < 0.001), gaze(r = 3.50, Cramer’s V = 0.13, p < 0.001), and taboo(r = 4.68, Cramer’s V = 0.13, p < 0.001). In contrast, in the images of citizen scientists generated by AI, the attributes with a higher numerical advantage include non-surreal elements (r = −9.42, Cramer’s V = 0.35, p < 0.001), non-gaze(r = −3.67, Cramer’s V = 0.13, p < 0.001), fluidity (r = −2.59, Cramer’s V = 0.07, p < 0.001), and non-taboo characteristics (r = −2.26, Cramer’s V = 0.13, p < 0.001).

This heatmap shows the standardized residuals of tertiary indicators (y-axis) within their secondary categories (x-axis) in the Resonate Dimension. Positive values (warm colors) indicate greater prevalence in scientist images, and negative values (cool colors) indicate greater prevalence in citizen scientist images. Surreal elements, gaze, and taboo-related cues appear more often in scientist images, whereas non-surreal elements, lack of gaze, fluidity, and non-taboo characteristics are more common in citizen scientist images.

## Discussion

### Identity work in the narrative dimension: The rhetorical blend of authority and accessibility

Image complexity refers to the structural intricacy of visual content, typically characterized by a higher density of pixel integration. AI-generated images of scientists tend to exhibit notably higher complexity, offering more detailed portrayals of scientific activities, environmental backgrounds, and visual structures. Such precise rendering highlights the depth and sophistication associated with scientific work and aligns with long-standing visual conventions that convey professionalism and authority in scientific domains. The large effect size accompanying this difference indicates that this visual advantage is not only statistically significant but also potentially meaningful in shaping how scientific professionalism is presented and interpreted within AI-mediated science communication contexts.

In the context of presence, AI-generated depictions of citizen scientists typically portray them as active practitioners situated naturally within outdoor or field-based environments. These images often employ medium-distance compositions that position citizen scientists within broader exploratory contexts, visually highlighting the participatory and open nature of their activities. This representational approach conveys qualities of accessibility and engagement that align with common narrative expectations of citizen science. In contrast, AI-generated images of scientists are predominantly situated in laboratory settings, reinforcing associations with scientific specialization and professional exclusivity. The medium-to-large effect sizes observed for these differences indicate that the model does not generate such distinctions incidentally but instead differentiates the two roles in a systematic manner, thereby contributing to their visual identity construction.

Regarding framing and composition, scientists are less frequently positioned at the visual center, whereas citizen scientists are more often depicted in central compositions. Central positioning, often associated with the aggregation of powerr [61], symbolizes stability and balance [62], which are closely linked to power distance [63]. In constructing the visual narrative of science, AI shifts the focus from authority to humanization, portraying scientists in a more approachable manner rather than emphasizing their centrality or authority. Although the effect sizes for these differences are relatively small, they nonetheless indicate subtle but consistent tendencies that support the narrative shift toward accessibility.

The visual grammar theory suggests that lateral view often evokes a sense of mystery or distance [53]. This is particularly evident in AI-generated depictions of scientists, which may perpetuate a sense of detachment between scientists and the public. The use of high-angle shots in scientific imagery further reinforces the perception of scientists as authoritative and aloof. Conversely, citizen scientists are depicted with a more relatable and accessible identity, aligning with their role as members of the general public. The medium effect sizes observed here support the argument that these visual strategies meaningfully contribute to perceived professional distance.

Color usage significantly influences viewer emotions and perceptions [64]. Bright, highly saturated colors are typically associated with joy, excitement, and positivity, while muted, low-saturation colors evoke calmness, introspection, or negativity [56]. Empirical findings suggest that color differentiation reinforces the identity work of science and citizen science. Scientists are often depicted in cool tones, which may convey a sense of abstraction or detachment, while citizen scientists are rendered in warmer tones, enhancing their relatability and vividness. Supported by moderate-to-large effect sizes, these color patterns suggest that chromatic differentiation functions as a consistent rhetorical device through which AI visually encodes the two identities.

Structural annotations, such as textual labels within images, provide contextual cues that highlight the situational and procedural dimensions of citizen science. Handwritten notes—for example, “July 12, 2023, during the third calibration of the instrument, I discovered…”—function as real-time records of scientific activity, lending these depictions a more accessible and human-centered quality. By reducing the sense of scientific mystique, such annotations frame science as a grounded, experiential practice rather than a strictly codified laboratory procedure. The medium-to-large effect sizes associated with these differences indicate that, within AI-generated imagery, structural annotations operate as a consistent narrative device that emphasizes the field-based and process-oriented portrayal of citizen scientists, whereas professional scientists are depicted through more institutionalized and standardized visual conventions.

### Act dimension role boundaries: The “professional” role of scientists and the “practical” role of citizen scientists

In the visual rhetoric of science communication, the contrasting representations of professional scientists and citizen scientists create a unique narrative tension. Professional scientists are often depicted through symbols such as silver hair, lab coats, and precision instruments, which construct their authority. Visual cues of age and experience reinforce public trust in their credibility. This imagery is rooted in traditional scientific narratives that celebrate “long-term dedication” and “knowledge transmission,” where even signs of fatigue become metaphors for devotion. The large effect sizes associated with professional clothing, scientific instruments, and knowledge symbols strengthen the argument that these visual motifs are central, not peripheral, to the construction of scientific authority in AI-generated imagery.

In the era of artificial intelligence, emerging visual narratives endow scientists with clearer facial textures and refined appearances—an idealized aesthetic that departs from the traditional “ascetic” persona often associated with scientific labor. From a narrative and visual structure perspective, scientists are frequently depicted working within laboratory settings, framed as “greenhouse flowers” nurtured by AI—that is, scientists portrayed as protected or insulated figures rather than exposed practitioners. They appear as observers sheltered by technology, conducting research in comfortable and secure environments. In contrast, citizen scientists are depicted through outdoor, practice-oriented activities, dressed in everyday clothing and using simple tools. The extremely large effect sizes observed in clothing and equipment further reinforce this binary: citizen scientists embody a grounded, practice-driven, and participatory identity that complements the professional depth associated with laboratory-based scientists.

The diversification of scientific narratives demands a reexamination of the boundaries of authority construction. While machine-augmented glasses extend the observational capabilities of scientists, the practical wisdom of citizen scientists expands the horizons of scientific understanding. Only by transcending the binary opposition between “white-coat authority” and “grassroots participation,” and by acknowledging the mutual enrichment of laboratory data and field experience, can we construct a more inclusive framework for science communication. This approach upholds the value of professional depth while unlocking the potential of public engagement, ultimately fostering the deep-rooted cultivation of scientific spirit within society.

### Interweaving of fantasy and reality in resonate: The fantasy narratives of scientists and the reality narratives of citizen science

Within AI-mediated science communication, the visual narratives of elite scientists and citizen scientists construct two distinct empathy landscapes. The imagery of elite scientists is often saturated with surreal symbols such as brain–computer interfaces and alienating laboratory settings. While these sci-fi narratives foster imagination about technological innovation, they risk creating emotional detachment by suspending the context of reality. The cold light refracted through laboratory domes and the floating chains of genetic diagrams abstract scientific practice into a futuristic philosophical proposition. The medium-to-large effect sizes for surreal elements underscore that this stylistic shift is not trivial but a notable pattern in AI’s imagination of elite science.

In contrast, the representation of citizen scientists is more firmly grounded in everyday life. Their images and activities are often situated in familiar settings, presenting scientific practice in a tangible and approachable manner. The large effect sizes associated with non-surreal elements highlight the salience of this groundedness, reflecting AI’s consistent depiction of citizen science as embedded within lived experience. This visual narrative reveals a stable tendency in AI-generated imagery to frame citizen science through everyday contexts, constructing a boundary of meaning that remains closely tied to the real world.

In the dimension of dynamism, citizen scientists redefine the interaction between science and the public through fluid and participatory practices. By integrating scientific activities into daily life—such as community observations, nature recordings, or online collaborative projects—they render science as a tangible “process.” Although the effect size for fluidity is small, the pattern complements the broader theme of participatory engagement. This approach dismantles the walls of the laboratory, transforming data collection and hypothesis testing into collective actions that the public can engage in. In contrast, AI often portrays elite scientists in static postures that reinforce symbolic systems of professional authority.

In the dimension of taboo, AI-generated depictions of elite scientists incorporate more elements of transgression, reflecting the contested and ethically complex nature of scientific research. These portrayals foreground scientific challenges and frontier innovations but also risk evoking ethical unease, inviting public reflection on the moral dimensions of science. The moderate effect sizes indicate that such imagery meaningfully contributes to ethical interpretation. By contrast, citizen scientists are framed through grounded, approachable activities that emphasize practical engagement. Yet this differentiated construction also reveals a potential narrative bias: AI consistently associates “scientists” with abstraction, risk, and taboo, while linking “citizen scientists” to safety and everyday familiarity. This pattern underscores the need to further examine how AI-mediated visual narratives may inadvertently reinforce binary distinctions in the public imagination.

## Research meaning

### Theoretical significance

This study offers a novel perspective in the field of rhetoric by introducing the concept of generative AI pragmatic experiments, emphasizing how generative artificial intelligence reconstructs scientific rhetoric. By exploring the role of AI in visual narratives, this research expands the dimensions of Visual Narrative Theory within science communication and provides a more refined theoretical framework for understanding the evolving landscape of science communication in today’s digital environment. This is particularly significant in the context of the rise of citizen science, as it highlights the interactivity and transparency of science communication, laying the groundwork for future research.

On an empirical level, the study reveals the complexity of rhetorical identity work by analyzing the visual representations of scientists and citizen scientists, thereby advancing our understanding of the relationship between authority and accessibility in AI-driven science communication. For instance, scientists often rely on professionalism and authority in their work, while citizen scientists gain trust and engagement through interaction and emotional connection with the public. This contrast between the role of scientists as experts and citizen scientists as practitioners underscores the diversity and flexibility in science communication. However, scientific authority is not solely dependent on the persuasiveness of logical reasoning; it is also deeply influenced by ethical values. By examining the relationship between scientists and citizen scientists, the study sheds light on the process of constructing scientific authority and reveals how different visual narrative rhetorical strategies in science and citizen science influence public understanding and participation. These insights provide a theoretical foundation for future research and communication strategies, emphasizing the need for science to be more closely integrated into the everyday lives of the public.

Specifically, scientists are often depicted working in laboratories, akin to “greenhouse flowers.” While this imagery highlights their professionalism and authority, it also situates them in a relatively closed and secure indoor environment, which may create a sense of distance between the public and scientific activities. This could inhibit opportunities for innovation and open dialogue, potentially reverting to the deficit model era [65], where scientific knowledge is seen as something to be transmitted unidirectionally from experts to a passive public.

### Practical significance

This study provides practical guidance on the application of generative artificial intelligence in scientific communication, emphasizing how visual narratives can enhance the effectiveness of information delivery. By flexibly utilizing AI technologies, science communicators can present complex scientific concepts in a more vivid and accessible manner, thereby increasing public understanding and engagement with scientific content. For example, employing engaging visual narrative frameworks, including fluidity, highlights the temporal and spatial movement of visual imagery, which can spark audience interest and reduce the sense of distance in scientific communication.

By analyzing the rise of citizen science and its challenges to traditional scientific discourse, this study offers solid theoretical support for encouraging public participation in scientific activities. It emphasizes the openness and inclusivity of science, prompting the public to engage more actively in scientific research and enhancing scientific education and community interaction. Such participation not only deepens public understanding and recognition of science but also cultivates a sense of social responsibility regarding science through shared practices and experiences, allowing more individuals to appreciate the application of science in daily life.

In addition, by exploring the image construction of AI for scientists and citizen scientists, it promotes a profound reflection on the nature of science and its social responsibility. By revealing potential ethical issues in science communication, such as the portrayal of scientists as surreal figures with taboo connotations by AI, this study calls for science communicators to exercise greater caution in visual narratives to avoid misleading the public. This emphasis on balancing scientific rigor with accessibility not only enhances the effectiveness of science communication but also reduces public misconceptions about science, thereby promoting more ethically conscious scientific communication practices.

### Limitation

Methodological Constraints: This study primarily relies on visual narrative analysis and pragmatic experiments, which somewhat limit the breadth and comprehensiveness of the research. Although these methods allow for an in-depth exploration of visual representations in scientific rhetoric, they may not encompass all variables that could influence scientific rhetoric. For instance, individual differences (such as age, educational background, or cultural factors), as well as changes in external social and political environments, could significantly affect audience understanding and responses. Therefore, these limitations may impact the generalizability of the conclusions, and future research should consider a more diverse array of methodologies to capture the multiplicity inherent in scientific communication.

Lack of Empirical Research on Persuasion Outcomes: While this study explores the application of Visual Narrative Theory in scientific communication, it does not empirically investigate the specific persuasive effects that may arise, particularly regarding how visual narratives influence public participation in science or enhance trust in science. Therefore, future research should aim to evaluate the persuasive effects of these different types of visual narrative strategies through empirical data, providing more practical insights. Moreover, scientists do not persuade others solely through logical reasoning; research has also shown that authority and the values it embodies play a significant role in the persuasive effects of scientific communication [66]. Many audience members may rely on the sense of authority and credibility conveyed by scientists, rather than solely on the data or evidence presented. Considering broader ethical and social factors in future studies will contribute to a more comprehensive understanding of the complexities and effectiveness of scientific communication.
